# Emerging Disease-Modifying Therapies in Neurodegeneration With Brain Iron Accumulation (NBIA) Disorders

**DOI:** 10.3389/fneur.2021.629414

**Published:** 2021-04-15

**Authors:** Vassilena Iankova, Ivan Karin, Thomas Klopstock, Susanne A. Schneider

**Affiliations:** ^1^Department of Neurology With Friedrich Baur Institute, University Hospital of Ludwig-Maximilians-Universität München, Munich, Germany; ^2^German Center for Neurodegenerative Diseases (DZNE), Munich, Germany; ^3^Munich Cluster for Systems Neurology, Munich, Germany

**Keywords:** neurodegeneration with brain iron accumulation, pantothenate kinase-associated neurodegeneration, plan, MPAN, BPAN, beta-propeller protein-associated neurodegeneration, disease-modification therapies, iron chelating agents

## Abstract

Neurodegeneration with Brain Iron Accumulation (NBIA) is a heterogeneous group of progressive neurodegenerative diseases characterized by iron deposition in the globus pallidus and the substantia nigra. As of today, 15 distinct monogenetic disease entities have been identified. The four most common forms are pantothenate kinase-associated neurodegeneration (PKAN), phospholipase A2 group VI (PLA2G6)-associated neurodegeneration (PLAN), beta-propeller protein-associated neurodegeneration (BPAN) and mitochondrial membrane protein-associated neurodegeneration (MPAN). Neurodegeneration with Brain Iron Accumulation disorders present with a wide spectrum of clinical symptoms such as movement disorder signs (dystonia, parkinsonism, chorea), pyramidal involvement (e.g., spasticity), speech disorders, cognitive decline, psychomotor retardation, and ocular abnormalities. Treatment remains largely symptomatic but new drugs are in the pipeline. In this review, we discuss the rationale of new compounds, summarize results from clinical trials, provide an overview of important results in cell lines and animal models and discuss the future development of disease-modifying therapies for NBIA disorders. A general mechanistic approach for treatment of NBIA disorders is with iron chelators which bind and remove iron. Few studies investigated the effect of deferiprone in PKAN, including a recent placebo-controlled double-blind multicenter trial, demonstrating radiological improvement with reduction of iron load in the basal ganglia and a trend to slowing of disease progression. Disease-modifying strategies address the specific metabolic pathways of the affected enzyme. Such tailor-made approaches include provision of an alternative substrate (e.g., fosmetpantotenate or 4′-phosphopantetheine for PKAN) in order to bypass the defective enzyme. A recent randomized controlled trial of fosmetpantotenate, however, did not show any significant benefit of the drug as compared to placebo, leading to early termination of the trials' extension phase. 4′-phosphopantetheine showed promising results in animal models and a clinical study in patients is currently underway. Another approach is the activation of other enzyme isoforms using small molecules (e.g., PZ-2891 in PKAN). There are also compounds which counteract downstream cellular effects. For example, deuterated polyunsaturated fatty acids (D-PUFA) may reduce mitochondrial lipid peroxidation in PLAN. In infantile neuroaxonal dystrophy (a subtype of PLAN), desipramine may be repurposed as it blocks ceramide accumulation. Gene replacement therapy is still in a preclinical stage.

## Introduction

Neurodegeneration with Brain Iron Accumulation (NBIA) comprises a heterogeneous group of disorders characterized by iron accumulation mainly in the globus pallidus and the substantia nigra, visible on MR imaging. These disorders present mostly with complex clinical syndromes defined primarily through extrapyramidal movement disorders (such as dystonia, chorea, parkinsonism), pyramidal symptoms, cognitive, optic and psychiatric abnormalities. The disease course is progressive, with early disability and decreasing quality of life.

NBIA disorders can show an autosomal recessive, autosomal dominant or X-linked inheritance pattern. They are considered ultrarare with a combined estimated prevalence of 1–9 per 1,000,000 (1). Currently, 15 genes have been identified underlying NBIA disorders. The four most common NBIA forms are pantothenate kinase-associated neurodegeneration (PKAN), phospholipase A2 group VI (PLA2G6)-associated neurodegeneration (PLAN), beta-propeller protein-associated neurodegeneration (BPAN) and mitochondrial membrane protein-associated neurodegeneration (MPAN). Dysfunctions in several pathophysiological pathways have been identified to be involved in NBIA disorders, including (1) coenzyme A biosynthesis, (2) lipid metabolism, (3) iron homeostasis, (4) autophagy and (5) other pathways of yet unknown function (2). This diversity in etiology and pathogenesis calls for different therapeutic approaches for the individual NBIA disorders. Currently only symptomatic therapies are available. Development of disease-modifying therapies is thus of great importance.

In this review, we summarize the current development of different mechanistic approaches for disease-modifying treatments. The emphasis is on iron chelation as a general approach. Disease-specific approaches are also discussed including (1) provision of alternative substrates downstream of the defect enzyme, (2) activation of isoenzymes, (3) counteracting downstream cellular effects, (4) gene therapies and (5) enzyme replacement therapies.

### Iron and Iron Chelation Therapies

Iron is a redox-active metal indispensable for cellular function. It is an essential cofactor for enzymes such as pyruvate dehydrogenase, helicases, ribonucleotide reductase and many more (3). Together with sulfur it builds iron-sulfur clusters, which are important components of metalloproteins such as Complex I, II, and III of the respiratory chain, which explains the crucial role iron plays in oxidative phosphorylation and energy production. It is furthermore essential for DNA synthesis and repair, phospholipid metabolism, and neurotransmitter synthesis (3).

Iron depositions in the brain are the eponymous feature and an important diagnostic biomarker for NBIA disorders. Despite that, only two NBIA forms—aceruloplasminemia and neuroferritinopathy—are caused by mutations in genes directly involved in iron homeostasis. Increased cellular iron in the brain has been described not only in NBIA disorders but also in normal aging and in multiple neurodegenerative diseases such as Parkinson's disease and Alzheimer's dementia (4–6). Iron dyshomeostasis with iron overload is associated with increased production of radical oxygen species and oxidative stress, protein misfolding and aggregation, dysfunction of the autophagy-lysosomal pathway, neuroinflammation and ferroptosis (iron-dependent apoptosis) (7).

In this context, Drecourt et al. recently presented compelling results from fibroblast cell lines derived from NBIA patients suggesting a uniform pathomechanism for the emergence of brain iron depositions (8). They ascribed the latter to post-translational abnormalities in the recycling of the transferrin receptor (TfR1) and the impairment of TfR1 palmitoylation. This offers new treatment targets (see below).

The origin and the role of iron depositions, however, in neurodegenerative diseases (NBIA and beyond) continue to be not fully understood. An important question remains unanswered: is iron only an epiphenomenon of disease pathogenesis or does it also accelerate neuronal death? In any case, brain iron depositions currently comprise an important target for potential disease-modifying therapies using iron chelators. Iron chelators are substances with a high affinity for iron, which aim to bind and remove the metal from different tissues in order to prevent or counteract iron accumulation. These have been used for the treatment of hematological diseases such as sickle-cell disease and thalassemia (9). NBIA disorders pose a challenge for iron chelating therapies. For successful iron removal from the brain, a substance ought to be able to cross the blood-brain barrier and to bind iron depositions in the basal ganglia without causing iron depletion in other brain areas or extraneural tissues.

Several iron chelators are available, the three main being deferiprone, deferasirox and desferrioxamine (also referred to as: deferoxamine). They differ in their chemical properties, pharmacokinetics and pharmacodynamics (10). Of these, deferiprone and deferasirox are administered orally. Deferasirox, however, has not shown sufficient reduction of brain iron depositions—potentially because of inefficient crossing of the blood-brain barrier (11), while deferiprone, thanks to the low molecular weight, favorable octanol-water partition coefficient and lipophilic properties, can successfully cross the blood-brain barrier and reach brain iron depositions (12, 13). The third chelator, desferrioxamine, has suboptimal oral pharmacokinetics and has to be administered subcutaneously or intravenously (10, 14). This leads to increased toxicity and reduced compliance of patients. Of the three mentioned substances, deferiprone is thus considered to be most potent and best suited for the treatment of NBIA syndromes.

#### Iron Chelation

##### Iron Chelation for PKAN

In the last decades, there have been several proof-of-concept case reports using chelating therapies in NBIA, which ultimately led to structured clinical trials. In the following, we briefly summarize the first exemplary case series and the main findings of the largest double-blind, placebo-controlled trial.

The first report was published in 1968 when Gallyas and Kornyey used desferrioxamine for the treatment of an NBIA disorder not further molecularly characterized (as prior to the genetic era) (15). There was no clinical improvement. Over the subsequent 40 years, case reports showed mixed and inconclusive results (7).

A milestone publication came from Zorzi et al. in 2011 who presented a phase-II trial in 9 PKAN patients treated with deferiprone. While there was no clinical benefit, the authors reported improvement of radiological markers (16). A similar trial included 6 NBIA patients (5 with genetically confirmed PKAN and 1 with idiopathic NBIA), treated for 36–48 months. Findings were heterogenous, including radiological improvement combined with clinical worsening or stabilization (17). In summary, still no definite conclusions could be drawn regarding the efficacy in NBIA.

This led us to do a placebo-controlled, double-blind, multicenter trial to study the efficacy and safety of deferiprone in PKAN patients; the results of which were recently presented (18). The study consisted of an 18-month treatment phase (deferiprone 30 mg/kg per day or placebo for 18 months) followed by a single-arm open-label extension phase (for another 18 months). In total, 86 gene-proven patients were included into the analysis. Iron concentration in the globus pallidus on MR imaging was significantly decreased in the deferiprone group. Clinically, during the double-blind phase, the study showed significant slowing of clinical progression by deferiprone in atypical PKAN patients compared to placebo. In classical PKAN, however, no significant slowing during the first treatment phase was observed. During the extension phase, patients from the deferiprone-deferiprone group showed no change in the rate of disease progression in comparison to the randomized clinical trial phase, while a significant slowing of progression was observed in patients who were switched from placebo to deferiprone.

Overall, this suggests that chelating therapy may be beneficial, at least in a subset of patients.

##### Iron Chelation in Other NBIA Subtypes

Most therapeutic studies on iron chelators in NBIA concentrate on PKAN, given it is the most frequent NBIA subtype. Individual treatment attempts in other NBIA subtypes will be reviewed separately in the following:

For BPAN, two patients treated with deferiprone have been published (19, 20). The clinical phenotype was typical in one and atypical in the other. Treatment for 4 months at a dose of 2,000 mg/day (30 mg/kg) and 12 months at a dose of 250 mg/day (3.85 mg/kg) failed to produce clinical benefit. In fact, it led to worsening in one case (with improvement after drug withdrawal) and both studies reported side effects (worsening of parkinsonism, abdominal pain, insomnia, restlessness, and agitation), so treatment was suspended. Comparative MRI imaging before and after iron chelation therapy was not performed in either of the two cases.

In MPAN, 2-year treatment in a 13-year-old led to reduction of iron content in the substantia nigra, while pallidal iron depositions remained unchanged and there was no clinical change (21). Another MPAN patient received deferiprone treatment which had to be discontinued because of gastrointestinal side effects (22).

In PLAN, treatment with deferiprone (at a dose of 30 mg/kg/d) led to increase of markers of iron metabolism such as transferrin, haptoglobin and hemopexin. However, clinical results were not reported (23).

In neuroferritinopathy, iron chelation therapy (desferrioxamine or deferiprone) in three patients which was combined with monthly venesections resulted in iron depletion in all. Clinically, there was worsening in one and no clinical change in the other two patients (24).

Aceruloplasminemia is a noteworthy example for the use of iron chelation treatment because there is systemic iron accumulation affecting the brain and visceral organs, e.g., liver and pancreas. The latter distinguishes aceruloplasminemia from other NBIA disorders, which present with pure brain iron overload.

Altogether, iron chelation in aceruloplasminemia has been reported in 51 cases in the literature (11, 25–66). Because of systemic iron overload, iron chelation substances were combined with other chelation strategies such as phlebotomy or fresh frozen plasma. A recent review discussed the use of different therapeutic approaches, i.e., monotherapy, combination of two types of iron chelation therapies, combination of iron chelation with zinc, vitamin C, vitamin E or consecutive use of iron chelation therapies (66). Overall, neurological effects of iron chelation therapies in aceruloplasminemia patients have been variable ranging from absent to moderate or good clinical improvement (7).

A valuable characteristic of this disorder is that systemic manifestations such as anemia and diabetes mellitus usually occur a decade prior to the onset of neurological symptoms (64). This allows pre-neurological diagnosis and long-term evaluation of potential preventive effects with respect to the development of brain iron accumulation and neurological symptoms. However, because of interindividual and intrafamilial variability precise prediction of the age at onset of brain-related symptoms is not possible. This makes interpretation of neuroprotective treatment effects challenging as a delayed or absent development of neurological features cannot be interpreted with certainty as result of preventive interventions.

According to our search, 26 cases of patients who were neurologically asymptomatic at the beginning of iron chelation treatment have been reported. Mixed results have been observed with some patients remaining asymptomatic. Relevant variations between type of chelation therapy, treatment and observation period should be noted. From the patients who remained neurologically asymptomatic at the end of the observation period, many were still in an age where neurological symptoms were not necessarily expected. Large studies with long-term follow-up would be needed.

One case with promising outcome was a male who presented in his mid-forties with mild resting tremor and brain iron accumulation on MR imaging (65). After 13 years of treatment with deferoxamine the patient had not developed further neurological symptoms or progression of radiological features. A second remarkable example is a neurologically asymptomatic woman diagnosed with aceruloplasminemia at the age of 62 because of anemia and a positive family history (65). She underwent intermittent phlebotomy over a period of 2 years, followed by 4 years of treatment with deferasirox. Over this time no development of neurological symptoms was observed. In summary, while reports about iron chelation treatment in neurologically asymptomatic patients with aceruloplasminemia need to be interpreted with caution, they support the idea of the potential application as a preventive measure in NBIA disorders.

As a further comment it needs to be added that many cases of aceruloplasminemia patients received deferoxamine (or sometimes deferasirox) rather than deferiprone, commonly used in PKAN, to treat the systemic iron overload. Furthermore, the chosen dose of deferiprone in aceruloplasminemia patients is usually higher—i.e., in other NBIA disorders deferiprone is administered at 20–30 mg/kg/day while in aceruoplasmienia the usual dose is more than twice as high, e.g., between 70 and 80 mg/kg/day (7).

In the aforementioned NBIA subtypes, besides PKAN, only individual treatment attempts with iron chelation have been described. No therapeutic trials with defined outcome measures have been performed and no quantitative data on treatment-related effects are available.

There are no reports of any iron chelation treatment attempts in patients with the other NBIA subtypes CoPAN, FAHN, Kufor-Rakeb disease, Woodhouse-Sakati syndrome, NBIA disorders caused by mutations in the genes *SCP2, CRAT, AP4M1, REPS1, and GTPBP2*.

##### What Conclusions Can Be Drawn for Iron Chelation as Treatment for NBIA?

(1) Negative predictive factors for the outcome of iron chelation treatment may be an early age of onset and advanced neurological abnormalities at the beginning of therapy. Patients with milder phenotypes and later onset tend to show better responses. (2) Despite the frequent lack of clinical improvement, slowing of progression and stabilization of the clinical condition have been observed in NBIA patients treated with deferiprone. (3) A discrepancy between radiological improvement (i.e., reduction of iron depositions on MR imaging) and the absence of clinical benefit is observed in many cases. One reason for this mismatch may be that in most of the patients neuronal damage was too far advanced and irreversible. Another explanation may be that iron is a secondary bystander, an epiphenomenon and not the cause of neurodegeneration. (4) Finally, studies in aceruloplasminema suggest a neuroprotective effect of iron chelation treatment when given early. However, this is difficult to implement for other NBIA subtypes (unless there is a positive family history leading to presymptomatic genetic testing) where CNS-related symptoms are already present at the time of diagnosis.

#### Activation of TfR1 Palmitoylation to Reduce Iron Overload

Transferrin receptor protein 1 (TfR1) is a transmembrane glycoprotein required for the endocytotic import of transferrin-bound iron into cells. Palmitoylation of TfR1 is a post-translational mechanism in healthy cells which inhibits TfR1-mediated endocytosis and thus reduces cellular iron intake. In NBIA cell lines (including *PANK2-, CRAT-, C19orf12-, PLA2G6*-mutant fibroblasts), a uniform impairment of TfR1 palmitoylation leading to disinhibition of iron import into cells was demonstrated as a key mechanism underlying brain iron accumulation (8).

Artesunate activates palmitoylation, facilitates internalization of TfR1 and thereby decreases iron uptake. Notably, it also reduces total iron content making it a drug candidate with potentially disease-modifying effects in NBIA.

To elaborate, artesunate is a first generation semisynthetic artemisinin derivate. Artemisinin is widely used in combination with other drugs to treat malaria, especially severe, multidrug-resistant forms (67). It also appears to have antineoplastic properties (68–70). Currently, artemisinin and its derivatives are being tested in different types of cancer (including breast cancer, renal cancer etc.). It has been suggested that the mechanism of action is based on increased iron content and increased TfR1 density on the surface of cancer cells which enable intracellular selective antineoplastic activation of artemisinin (71, 72).

Artemisinin and its derivates are characterized by an endoperoxid bond (ROOR'). Iron, heme or heme-bound proteins lead to splitting of this bond and the formation of radicals and reactive oxygen species with subsequent cell damage and apoptosis (71, 72).

It has been debated whether an increase of artemisinin-induced oxidative stress and cytotoxicity in cells with increased iron metabolism is indeed the right approach for NBIA disorders where neuronal cell damage must be prevented and not accelerated.

Furthermore, some of the functional mechanisms of artemisinin and its derivatives overlap with the pathophysiological abnormalities already seen in NBIA disorders which may reinforce cellular damage. For example, they induce lipid peroxidation and increase oxidative stress which is an important part of the pathomechanism in PLAN (71). They also increase ER stress, an important mechanism in BPAN (71, 73).

To date, there are no data of the use of artesunate in NBIA patients. Prior to its use further preclinical studies are needed.

### Disease-Specific Treatments Based on Molecular Mechanisms of the NBIA Subtype

In recent years, disease-specific treatments have been developed which will be reviewed and discussed in the following (see Table 1).

**Table 1:** Therapeutic strategies beyond iron chelation.

	**Substance**	**Mechanism of action**	**Stage of development**	
			**Preclinical phase**	**Clinical study, RCT Number, main outcome**
All NBIAs	Artesunate	Activation of TfR1 palmitoylation	Cell culture (8)	–
PKAN	Coenzyme A	Direct supplementation	Cell culture, *Drosophila melanogaster, Caenorhabditis elegans* (8, 76–78)	–
	Pantothenate	Increase of physiological substrate	Cell culture, Drosophila melanogaster, mouse model (78–80)	–
	Fosmetpantothenate	Alternative substrate	Cell culture (139)	Phase III (86–88) RCT Number: NCT03041116 No difference in meeting the primary and secondary outcome measures between the treatment and the placebo group, extension phase terminated early
	4′-Phosphopantetheine	Alternative substrate	Mouse model (80)	Phase II (89) RCT Number: NCT04182763 currently recruiting
	Pantethine	Alternative substrate	Mouse model (90), *Drosophila melanogaster* (78)	Phase II (92) No improvement of motor symptoms
	S-Acetyl-4‘-Phosphopantetheine	Alternative substrate	Cell culture, *Drosophila melanogaster* (91)	–
	Pantazine	Isoenzyme activation	Cell culture, mouse model (93)	–
	VTAC 1-9	Isoenzyme activation	Announced. No data published yet (94)	–
	AAV9-mediated gene therapy	Gene supplementation therapy	Mouse model. Announced. No data published yet (95)	–
PLAN	D-PUFAs	Inhibition of lipid peroxidation	Cell culture, *Drosophila melanogaster* model (97)	Phase II/III (109, 110) RCT Number: NCT03570931 Still active, no results yet
	Desipramine	Reduction of ceramide accumulation	*Drosophila melanogaster* model (111)	Phase IV (112) RCT Number: NCT03726996 Still active, no results yet
	AAV9.hSyn1.hPLA2G6	Gene supplementation therapy	Mouse model (113)	–
	*C19orf12 Overexpression (no substance)*	*Overexpression of mitochondrial membrane-associated protein*	*Drosophila melanogaster model (98)*	–
BPAN	Rapamycin (mTOR inhibitor)	Autophagy activation	Mouse model (119)	–
	TUDCA	ER stress inhibition	Mouse model (119)	–
MPAN	Testing of potential compounds is underway			
Aceruloplasminemia	Ceruloplasmin	Enzyme replacement therapy	Mouse model (134)	–

*RCT, registered clinical trial number; RCT, registered clinical trial number; NBIA, Neurodegeneration with brain iron accumulation; PKAN, Pantothenate kinase-associated neurodegeneration; PLAN, Phospholipase A2 group VI (PLA2G6)-associated neurodegeneration; BPAN, Beta-propeller protein-associated neurodegeneration; MPAN, Mitochondrial membrane protein-associated neurodegeneration; TfR1, Transferrin receptor protein 1; D-PUFA, Deuterated polyunsaturated fatty acids; AAV9.hSyn1.hPLA2G6, Adeno-associated virus vector with a healthy PLA2G6 copy; mTOR, mammalian target of rapamycin; TUDCA, Tauroursodeoxycholic acid*.

#### PKAN

PKAN is caused by a mutation in the *PANK2* gene on chromosome 20 which encodes the pantothenate kinase 2 enzyme. This is the only enzyme isoform located in mitochondria. It catalyzes the first step in the biosynthesis of coenzyme A (CoA), namely the phosphorylation of pantothenate to 4′-phosphopantothenate. CoA plays a key role in multiple metabolic processes such as but not limited to the citric acid cycle, fatty acid oxidation, aminoacid metabolism and neurotransmitter synthesis.

In PKAN, two clinical phenotypes are often distinguished—classic and atypical (74, 75). Dystonia is an important clinical feature, typically affecting the lower limbs, but orolingual involvement is also common and points away from many other dystonic disorders (such as DYT1 dystonia). The atypical phenotype differs from the classic variant by later onset, slower progression and a milder phenotype.

Because of its relative frequency among the NBIA disorders, most clinical trials were conducted in PKAN. Likewise, PKAN has been the main focus of research for the development of disease-modifying therapies in cell cultures, animal models and humans.

An important therapeutic strategy for PKAN is the utilization of alternative substrates to bypass the PANK2 enzymatic defect and enable CoA biosynthesis. Intermediate substances – such as but not limited to fosmetpantotenate and 4′-phosphopantetheine—as well as direct CoA supplementation have been tested.

##### Coenzyme A—The Direct Supplementation Approach

CoA administration demonstrated several beneficial effects in preclinical NBIA models: Thus, CoA supplementation improved neuronal viability, neuronal excitability and firing activity, decreased ROS levels and recovered mitochondrial function in human-derived pluripotent stem cells converted to PKAN neurons (76). CoA rescued phenotypes caused by CoA depletion in cultured cells, as well as in Caenorhabditis elegans and Drosophila melanogaster models (77). CoA also restored the cell count of PANK-depleted cells in a PKAN D. melanogaster model, albeit it was less effective and more toxic than pantethine (78). Interestingly, CoA supplementation increased TfR1 palmitoylation (the role of which was discussed above) in cultured fibroblasts with *PANK2* mutations (8).

However, CoA was found to be unstable (77): After 30 min, 90% of CoA was degraded in serum-containing medium. Furthermore, CoA is incapable of passing through membranes. Instead, CoA is quickly converted into its precursor substrate, i.e., 4′-phosphopantetheine, which is stable, can be measured in plasma hours after the supplementation and passes through cell membranes of cultured cells (77).

An NBIA variant metabolically closely related to PKAN is CoA synthase (COASY) protein-associated neurodegeneration (CoPAN) due to a defect in the biosynthesis of CoA, caused by mutations in the *COASY* gene. In the metabolic cascade, the encoded COASY protein is located downstream of PANK2 and catalyzes the last two steps of CoA biosynthesis. Preclinical studies in *COASY* mutant flies and cultured cells showed that there is no significant improvement by CoA supplementation (77). The above-mentioned problems of CoA instability are particularly limiting in the case of CoPAN because for the intracellular conversion of the precursor substrate into CoA the defect enzyme COASY would still be needed.

In summary, exogenously supplemented CoA is unstable in human serum and gets hydrolyzed and converted to the precursor substrate. CoA supplementation itself is thus not helpful for the treatment of CoPAN. With regard to PKAN the following question arises: If CoA supplementation can rescue the clinical phenotype of PKAN but is unstable and gets converted to its precursor substrate in order to enter the cells, would a therapy with CoA still be useful or is a direct approach with 4′-phosphopantetheine (section 4′-Phosphopantetheine—Another Alternative Substrate) a superior option?

##### Pantothenate—To Increase the Physiological Substrate

Pantothenate is the substrate of the PANK2 enzyme. Cultured fibroblasts derived from patients with different *PANK2* mutations show altered cell morphology, accumulation of iron and lipofuscin, increased oxidative stress as well as increased mitochondrial lipid peroxidation (78). In a cell experiment, PKAN and control fibroblasts were treated with pantothenate in increasing concentrations (79). Two mutant fibroblast cell lines with residual PANK2 enzyme activity were responsive to pantothenate which was able to stabilize PANK2 enzyme expression, recover all pathological abnormalities and protect against induced cell death. On the contrary, pantothenate did not produce positive effects in a cell line with practically absent PANK2 enzyme expression levels. This is in line with studies in PKAN Drosophila melanogaster and mouse models with absent PANK2 enzyme activity: In these, pantothenate also did not produce any benefit (78, 80).

These results suggest that high dose pantothenate may be potentially useful in PKAN patients with residual PANK2 enzyme activity. However, there is scarce data from pantothenate in patients. In a patient with HARP syndrome (hypoprebetalipoproteinemia, acanthocytosis, retinitis pigmentosa, and pallidal degeneration) due to *PANK2* mutations, administration of pantothenic acid at a dose of 2 g a day did not lead to any clinical or laboratory change (81). However, it was not clear from the report in how far the mutations were associated with residual enzyme activity. Similarly, in our own experience pantothenate as a dietary supplement does not produce clinically relevant effects.

##### Fosmetpantotenate—An Alternative Substrate

As mentioned above, PANK2 converts pantothenate to 4′-phosphopantothenate. Attempts to substitute 4′-phosphopantothenate failed because of an insufficient ability to pass through cell membranes (82). Consequently, fosmetpantotenate (also known as RE-024, Retrophin, Inc.) was developed which crosses the blood-brain barrier and neuronal cell membranes (83). Intracellularly, it is metabolized to 4′-phosphopantothenate thus providing a substrate for the CoA biosynthesis and bypassing the defect of the PANK2 enzyme.

In 2017, some clinical improvement (reflected by various clinical measures) was reported in case reports of an atypical PKAN patient treated with 2.64 mg/kg/day fosmetpantotenate for 12 months (84) and of two PKAN siblings treated at a dose of 3 mg/kg/day for 47 weeks (85).

This led to the initiation of a randomized, double-blind, placebo-controlled phase III clinical trial testing the efficacy and safety of fosmetpantotenate in PKAN (86). In total, 84 patients were randomized to receive the active drug or placebo three times daily for 24 weeks. However, there was no difference between the two groups in meeting the primary (change in PKAN-ADL) or secondary (UPDRS part III score) outcome measures (87). Accordingly, the open-label extension phase was terminated early and the sponsor discontinued the drug development program for this compound (88).

##### 4′-Phosphopantetheine—Another Alternative Substrate

4′-Phosphopantetheine is another substrate which bypasses the PANK2 enzyme defect and can be catalyzed by COASY to 4′-dephospho-CoA and subsequently to CoA.

Experiments in *PANK2* knockout cell and mouse models demonstrated positive effects (80). In fibroblasts derived from PKAN patients, 24 hours of treatment with 4′-phosphopantetheine led to recovery of the expression of *COASY* and *Tfrc* (80). In mice, 14-day treatment with 4′-phosphopantetheine was able to normalize the expression of *COASY*, iron homeostasis genes *(Tfrc, Ireb2, Drd1)* and dopamine markers and led to recovery of protein activity of pyruvate dehydrogenase and complex I of the respiratory chain (80). An important limitation of this experiment was, however, that *PANK2* knockout mice displayed mild late-onset retinopathy as the only feature, without any neurological symptoms. Thus, the model does not fully recapture the clinical picture seen in PKAN patients, so the effects on neurological features remain unclear.

To test the effect in humans, a compound called CoA-Z (not to be mistaken with the enzyme COASY) was developed. It contains 4′-phosphopantetheine as the active ingredient. A first in-human study was performed to examine the safety and tolerability of the drug as well as the pharmacodynamic profile of *COASY* mRNA exression (89). Six PKAN patients (three with atypical and three with classic PKAN) were enrolled and received a 14-day treatment followed by a 14-day washout phase. No adverse events and side effects were reported. An increase in *COASY* mRNA expression in blood was observed in both groups of PKAN patients.

Recently, a clinical trial investigating CoA-Z was initiated at Oregon Health and Science University, USA. The study is planned as a 6-month dose-ranging, randomized, double-blind, placebo-controlled trial. Patients will be randomized into one of four groups treated with high, medium or low dose CoA-Z or placebo. The protocol entails a single-arm open-label extension phase currently expanded to 30 months at the medium assigned dose of CoA-Z.

The trial is actively recruiting. Currently, 60 patients are expected to be enrolled (89). Northern American PKAN patients (from the US or Canada) from all age groups (3 months−89 years) are eligible to participate in the study. Further details can be found on https://clinicaltrials.gov/ct2/show/NCT04182763. Efforts to expand the clinical trial to the Netherlands and the United Kingdom are currently underway.

##### Pantethine—Another Alternative Substrate

Pantethine is a derivative of pantothenic acid. Enzymatically it can be broken down into two pantetheine molecules which can subsequently be phosphorylated to 4′-phosphopantetheine thus providing a substrate for CoA biosynthesis. Preclinical work on *PANK2* knockout models showed the compound can rescue the clinical phenotype in mice (90) and recover CoA levels and mitochondrial function in Drosophila melanogaster (78). An instability of pantetheine in serum due to pantetheinase activity has been discussed as a limiting factor in previous work (91).

Pantethine is currently used as a nutritional supplement in patients with hyperlipidemia. A single-arm open-label clinical trial with pantethine was performed in 15 patients with PKAN (92). A 24-week treatment with a dose of 60 mg/kg per day did not lead to improvement of the motor symptoms. A possible delay of motor function worsening was discussed.

##### S- Acetyl-4′-Phosphopantetheine—Another Alternative Substrate

Another compound under development is s-acetyl-4′-phosphopantetheine, an acylated form of 4′-phosphopantetheine. In preclinical studies, it was found to be stable in serum and to have the ability to rescue phenotypes associated with PANK deficiency (91).

No studies on patients have been conducted.

##### Pantazines—Activation of Isoenzymes

Another disease-specific approach being tested in PKAN is the activation of PANK enzyme isoforms using small compounds called pantazines (93). A potent pantazine is PZ-2891 which functions as an allosteric activator of PANK3 by occupying the pantothenate pocket.

Physiologically, the PANK3 enzyme can be found in two different conformations—an active one, stabilized by an ATP-Mg2+ complex, and an inactive one, stabilized by acetyl-CoA. The latter operates as a physiological feedback inhibitor. When PZ-2891 binds PANK3, it stabilizes the active form and prevents it from binding acetyl-CoA so that the PANK3 activity becomes refractory to acetyl-CoA inhibition.

Administration of PZ-2891 increased CoA levels in cultured cells and in mouse liver and brain (93). A knockout mouse model of brain CoA deficiency which exhibited weight loss, severe locomotor impairment and early death improved following PZ-2891 therapy. Treated mice showed weight gain, significant increase in median survival from 52 to 150 days, improved locomotor activity and elevation of CoA levels in the brain.

There are no studies in patients so far.

##### VTAC 1-9—Activation of Isoenzymes

Further compounds called VTAC 1-9 have been found to activate a PANK isoenzyme (94). Their further development is underway. No preclinical data have been published yet.

##### PANK2 Gene Therapy

At this year's International Symposium on NBIA and related disorders, Hayflick and colleagues announced that they are working on a gene therapy for PKAN using AAV9 constructs containing the full-length human *PANK2* gene (95). In their preclinical studies, mice receive stereotactic injections targeting the globus pallidus. Results were not presented, as the project is still in its early stages.

##### What Have We Learned From Research Efforts in PKAN?

Clinical research to find disease-modifying treatment approaches for PKAN has been quite active in the last decade. It concentrated on two main strategies—(1) the use of iron chelators (discussed in section Iron and iron chelation therapies) and (2) the use of alternative substrates bypassing the PANK2 enzyme defect. Increase of the physiological substrate pantothenate, showed no positive results in single case reports. Direct supplementation of Coenzyme A had shown promising results in preclinical models despite its instability in human serum. Fosmetpantotenate failed to produce a significant effect in a placebo-controlled clinical trial in patients. Currently, 4′-phosphopantetheine is being tested in patients with PKAN. A pilot study with pantethine did not show any clinical improvement. S-Acetyl-4′-Phosphopantetheine is another compound under development. The activation of isoenzymes is a further strategy used in preclinical models. Further studies are needed. The development of PANK2 gene therapy is in its early stages.

#### PLAN

PLAN comprises a group of disorders caused by mutations in the *PLA2G6* gene on chromosome 22, which encodes an intracellular, calcium-independent phospholipase A2 group VI. This enzyme plays a key role in phospholipid metabolism and selectively hydrolyses the sn2 ester bond in glycerophospholipids (96). This results in the release of free polyunsaturated fatty acids and lysophospholipids. Studies in preclinical models have shown disturbances in phospholipid metabolism as well as damage in the mitochondrial inner membrane (97). In an *iPLA2-VIA*-deficient Drosophila melanogaster model, reduction of phospholipid acyl chains length was observed which led to ER stress and facilitated alpha-synuclein aggregation (98).

PLA2G6-associated neurodegeneration (PLAN) comprises different clinical phenotypes—infantile neuroaxonal dystrophy (INAD), atypical neuroaxonal dystrophy (ANAD) and dystonia-parkinsonism (99). Neuroaxonal dystrophy refers to the neuropathological finding of focal granular enlargements within axons (so-called axonal spheroids), seen in biopsies from INAD and ANAD patients (100).

As the name suggests, INAD presents early in life, mostly in the 1st year of life. A recent natural history study showed developmental delay in 67% of patients (101). In total, 37% of patients never learned to walk independently. All patients experienced consecutive psychomotor regression.

Further symptoms of INAD are ataxia, early truncal hypotonia followed by spastic tetraparesis, neuroophthalmologic abnormalities with rapid progression, and loss of ambulation within 5 years. The most frequent imaging finding is cerebellar atrophy. In addition, 14 of 29 (48%) INAD patients presented with brain iron accumulation on MR imaging (102). Further imaging findings have also been described (103).

ANAD is broadly considered as a varying phenotype, which diverges from the classical INAD phenotype in terms of age of onset and disease progression presenting with later onset (median 4 years) and slower progression. Symptoms reported in ANAD include ataxia, gait disability, dystonia, dysarthria and spastic paresis.

A subset of PLAN patients presents with early-onset (before age 40 years) parkinsonism which can be combined with dystonia, gait abnormalities, neuropsychiatric abnormalities and cognitive decline (dystonia-parkinsonism variant, PARK14).

In all these phenotypes, brain iron accumulation in the basal ganglia may be present but is no obligatory feature. Thus, strictly speaking, not all PLAN patients fulfill the criteria of NBIA.

Regarding the development of disease-modifying therapies for PLAN, scientists have concentrated mostly on INAD. Here we present recent highlights in drug development:

##### Deuterated Polyunsaturated Fatty Acids—Slowing Down Lipid Peroxidation

The brain is rich in polyunsaturated fatty acids (PUFAs) which are part of lipid membrane layers and help reduce oxidative stress (104, 105) by preventing the accumulation of lipid peroxides and reducing the accumulation of ROS.

Deuterated PUFAs were found to restore mitochondrial membrane potentials in cultured fibroblasts (97). In a Drosophila melanogaster *PLA2G6* knockout model D-PUFAs were able to partially restore locomotor function (97).

The effect of D-PUFAs is currently being tested in patients with a variety of neurodegenerative and neuromuscular disorders such as Friedreich's ataxia, late-onset Tay-Sachs and progressive supranuclear palsy. In a recent phase I/II, double-blind, placebo-controlled clinical trial in Friedreich's ataxia, the drug RT001 was well-tolerated with diarrhea being the only treatment-related adverse event (106).

The use of D-PUFAs, i.e., RT001, in NBIA has also been reported: A 2-year-old INAD patient improved on 3.6 g/day RT001 (107) demonstrating overall improvement, especially of bulbar and ocular functions, and disease stabilization after 1 year of treatment (108). A second case, a 5-year-old boy, showed initial improvement of fine motor skills, attention and social interaction, however, effects did not last, so treatment was discontinued after 6 months (108).

Currently, a prospective, single-arm, open-label clinical study is being conducted in two centers in the United States—one in California and one in New Jersey—to test the efficacy and safety of RT001 in patients with INAD (109). Patients are treated with RT001 3.84 g/day for a period of 12 months with a possible 12-month extension phase. In total, 19 patients were enrolled. (Clinical Trials No: NCT03570931) Completion of the 12-month extension phase is expected for August 2021 (110). No results or other details have yet been published.

##### Desipramine—Reduction of Ceramide Accumulation

Phospholipids such as sphingomyelin and ceramide are crucial elements of the plasma membrane and play an important role in cell signaling. The conversion of ceramide phosphoethanolamines (CPE) and sphingomyelin to ceramide is catalyzed by the acidic sphingomyelinase (111). This step is inhibited by desipramine which is widely used as a tricyclic antidepressant.

Reduced levels of CPE were found in a PLAN loss-of-function fly model. This was accompanied by increase in ceramide with lysosomal accumulation (111) with subsequent cell membrane defects and disturbances in endocytosis. Desipramine treatment resulted in reduction of ceramides and alleviation of lysosomal stress and suppression of neurodegeneration.

A clinical trial at Duke University examining the off-label use of desipramine in INAD patients was completed in August 2019 (112). Four patients were enrolled. No results are yet available.

##### Gene Replacement Therapy

Adeno-associated viruses can be used as vectors for delivery of a gene. Somatic gene replacement therapy was recently tested in a knock-in mouse model of PLAN (113). The phenotype of the untreated PLA2G6-INAD mouse was characterized by early motor incoordination, muscle atrophy with weakness and gait impairment, and reduced lifespan. Pathological examination revealed widespread neuronal loss, gliosis and microglial activation in brain and spinal cord.

A single dose of AAV9.hSyn1.hPLA2G6 (an AAV-vector with a healthy *PLA2G6* gene copy), administered pre-symptomatically, led to significant weight gain, prevention of motor decline and extension of lifespan. Significant improvement of neuronal viability as well as amelioration of neurodegeneration in the brain were also reported. Neuronal cell counts, however, remained significantly lower than in wild-type mice and the level of neuroinflammation (astrocytosis and microglial activation) remained unchanged.

A study in patients with *PLA2G6* mutations is in preparation.

##### Pharmacological Chaperons—Stabilizing Protein Folding of Mutant PLA2G6 Proteins

Preclinical work on the development of therapies aiming at improving the function of mutant PLA2G6 proteins is being carried out (114). The goal is to identify compounds functioning as pharmacological chaperons by stabilizing protein folding in mutant PLA2G6 proteins. Compound library screens are planned.

##### Overexpression of C19orf12

Recently, a close relationship in the pathogenesis of PLAN and MPAN was proposed when neuronal overexpression of the MPAN-associated *C19orf12* gene homolog CG3740 improved markers of disease in an *iPLA2-VIA-*deficient Drosophila melanogaster model. In detail, *C19orf12* overexpression rescued motor abnormalities, reduced bang-sensitivity and improved the viability of dopaminergic neurons (98). Furthermore, it suppressed brain vacuolar formation, decreased the proportion of phospholipids with shortened acyl-chain length and ameliorated ER stress and alpha-synuclein aggregation.

There is no clinical application of this yet.

##### What Have We Learned From Research Efforts on PLAN?

A general approach for complex neurodegenerative disorders is to understand the cascade of neurodegeneration and to identify cellular targets. The approach has long been to interfere with downstream mechanisms which ideally act as a one-size-fits all ailment. In the context of PLAN, most compounds indeed are not disease-specific but tackle targets rather downstream in the cascade of neurodegeneration. In addition, a gene therapy program is in the pipeline for patients with *PLA2G6 mutations*. However, challenges regarding the ideal route of administration have to be overcome.

#### BPAN

BPAN is characterized by childhood-onset seizures and developmental delay with loss of expressive language skills, stereotypies, behavioral abnormalities and intellectual disabilities. As the disease progresses, in adulthood, patients develop movement disorders (e.g., dystonia and parkinsonism).

Neuropathological examination in BPAN revealed mixed pathology with significant nigral neuronal loss and gliosis, and iron accumulation. Wide-spread hyperphosphorylated tau depositions in form of neurofibrillary tangles, pre-tangles and neuropil threads containing both 3-R and 4-R tau isoforms have been observed (115). Alpha-synuclein depositions have not been reported.

BPAN is caused by *de novo* mutations in the *WDR45* gene located on the X chromosome. It encodes a WD40 repeat-containing phosphoinositide-interacting protein 4 (WIPI4) which is involved in the early stages of autophagy (116). Numerous genes are involved in autophagy regulation. This includes WIPI4, one of the four mammalian homologs of the autophagy-related protein Atg18 found in yeast. Decreased autophagy activity and accumulation of ATG9A-positive structures have been reported in lymphoblastoid cell lines derived from BPAN patients suggesting impairment of the autophagosome formation (117).

Different knockout mouse models reflecting the BPAN phenotype have been developed (118), including a recently presented model generated by a germline mutation (119). These exhibit poor motor coordination, greatly impaired learning and memory, and extensive axon swelling with numerous axon spheroids.

Pharmaceutical rescue attempts for BPAN are based on the activation of autophagy and the inhibition of ER stress.

##### Activation of Autophagy

Mechanistic Target Of Rapamycin (mTOR) is a protein kinase which inhibits autophagy by phosphorylating Ulk1, a kinase required for the initiation of autophagy (120). mTOR plays an important role in the regulation of cell growth, lipid and nucleotide synthesis and biogenesis of organelles (such as lysosomes and ribosomes) (121). It regulates the adaptation of the organism to feeding and fasting and is sensitive to multiple upstream factors including nutrients, growth factors and stressors. Anti-aging properties have also been hypothesized.

Rapamycin inhibits mTOR and leads to disinhibition of autophagy. It is utilized as an immunosuppressant after organ transplantation. Current studies explore potential effects for different types of cancer (122, 123). For neurodegenerative diseases, rapamycin is also a potential candidate after preclinical studies showed neuroprotective effects in animals with decreased dopaminergic cell death in the substantia nigra in a model of Parkinson's disease and improvement of cognitive deficits in models of Alzheimer's dementia (124). Improved autophagic clearance is thought to be the mechanism of action.

In a BPAN mouse model, rapamycin was able to reduce ER stress, partially restore autophagy and alleviate neuronal death. No effect on ER expansion was observed (119).

The efficacy of mTOR inhibition is being currently tested in patients with (genetic) Parkinson's disease (125, 126). We are not aware of trials in BPAN.

Independently, Dr. R. Ketteler and his team at UCL have performed screening of compounds in BPAN cell lines and have identified potential molecules able to restore autophagy (127). Further testing of these potential therapies in a BPAN tissue model is currently planned.

Furthermore, Dr. H. Zhang recently reported results on the role of the *WDR45* gene product in autophagosome-lysosomal fusion (128). He discussed activation of autophagosome-lysosomal fusion (e.g., through the inhibition of O-GlcNAcylation of the SNARE protein SNAP-29) as a potential therapeutic strategy for restoring autophagic defects in BPAN.

##### ER Stress Inhibitor

Tauroursodeoxycholic acid (TUDCA) is in very early stages of development. The compound is an ER stress inhibitor which reduced ER stress and rescued cellular death in *WDR45*-deficient cells. However, no improvement of apoptosis in neurons and ER expansion was observed (119).

##### What Have We Learned From Research Efforts on BPAN?

Compared to PKAN, research in BPAN is still in early stages. Thus, there are no trials in patients yet. Preclinical research suggests the potential use of mTORC1 inhibitors such as rapamycin, which is likely to be a non-specific approach. The induction of autophagic flux has been hypothesized to be a crucial mechanism of counteracting neurodegeneration and if beneficial, could also be implemented in other neurodegenerative disorders.

#### MPAN

MPAN is a monogenic NBIA disorder caused by mutations in the *C19orf12* gene on chromosome 19. The clinical picture is characterized by pyramidal involvement (e.g. spasticity), extrapyramidal symptoms (e.g. dystonia), cognitive and psychiatric abnormalities as well as neuroophthalmologic involvement (129).

The *C19orf12* gene is thought to encode a mitochondrial membrane protein which is located in mitochondria, in the ER and in the contact zones, respectively, so called mitochondria-ER associated membranes (MAM) (130). MAMs are crucial for the regulation of calcium, lipid transfer, mitochondrial fission and autophagosome assembly. MAMs have also been associated with other neurodegenerative disorders such as Alzheimer's dementia (131, 132).

In MPAN, mis-localization of the C19orf12 protein leads to increase of Ca2+ in mitochondria with increased susceptibility to oxidative stress as shown in fibroblasts derived from MPAN patients (130). While the exact function of the *C19orf12* gene product is not known, it has been hypothesized that it may play an important role in autophagy of defect mitochondria.

Animal models have been developed to further investigate the underlying pathophysiology. A Drosophila melanogaster model with downregulation of both orthologous genes of *C19orf12*—*CG3740* and *CG11671* recapitulates the human phenotype, i.e., flies show climbing abnormalities, reduced survival and vacuolation in brain (133). Further preclinical studies including testing potential therapies in flies and in patient cell lines are underway.

##### What Have We Learned From Research Efforts on MPAN?

The development of disease-modifying treatment strategies for MPAN is in very early stages. Interesting preclinical models have enabled to gain more insight into the function of the C19orf12 protein and its importance for mitochondria-ER associated membranes. A connection between the pathophysiology of PLAN and MPAN was also demonstrated which is an important step in understanding these subforms and may set the ground for development of related therapies.

#### Aceruloplasminemia

Aceruloplasminemia due to mutations in the *ceruloplasmin* (*CP*) gene on chromosome 3 is characterized by, as mentioned above, iron accumulation not only in the brain, but also in other organs including liver and pancreas. Key symptoms are diabetes mellitus, retinal degeneration, anemia, and neurological symptoms such as cerebellar ataxia, movement disorders and behavioral changes.

The encoded protein, ceruloplasmin, is a metalloprotein with copper-dependent ferroxidase activity which catalyzes the oxidation of Fe2+ to Fe3+. Ceruloplasmin carries the large majority of the total copper in plasma. It also enables transportation of iron in plasma.

Results from iron chelation therapies in patients with aceruloplasminemia are discussed above in section Iron and iron chelation therapies. A disease-specific therapeutic approach may be the use of enzyme replacement therapy. Treatment of a *ceruloplasmin*-knockout mouse model with ceruloplasmin (administered intraperitoneally) led to significant improvement of motor coordination, complete recovery of the CP-associated ferroxidase activity in the brain and rescue of Purkinje cells. Iron content was reduced in the brain and the choroid plexus but not in the liver (134).

There are no data of the use in patients. Gene therapy has not been addressed so far.

##### What Have We Learned From Research Efforts on Aceruloplasminemia?

Given the nature of the disease with onset in adulthood, aceruloplasminemia has enabled researchers to test the role of iron chelators in neurologically presymptomatic patients (see section Iron Chelation in Other NBIA Subtypes), albeit it cannot be proven in how far a mild disease course is causally linked to potentially preventive interventions. Preclinical experiments suggest that enzyme replacement therapy may be another therapeutic strategy but there is no data from patients.

## Discussion

With increasing insights into rare genetic disorders, precision medicine becomes increasingly realistic. In this review, we summarized emerging disease-modifying therapies for NBIA disorders.

Generally, the major challenge in the development of disease-specific treatments for complex diseases is the lack of understanding the pathogenesis. In the last decade, basic research has provided valuable insights into some of the NBIA disorders, which have paved the way for new treatment avenues. Yet, further work to unravel the function of the defined gene products is still needed. In this regard development of suitable animal models which reflect the biochemical, neuropathological and clinical abnormalities of each disorder have been another challenge. Thus, while a mouse model is now available for PKAN, PLAN, and BPAN, there is no rodent model for MPAN where research is limited to fly models.

As summarized in this review, treatment of NBIA now goes beyond symptomatic removal of iron and now includes compounds to bypass the effected metabolic pathways (by using alternative substrates), activation of isoenzymes, overexpression of the affected gene or activation of autophagy.

One study is currently actively recruiting: for PKAN testing 4′-phosphopantetheine-containing CoA-Z. Other compounds are still in the preclinical phase of development.

It will be interesting to see what other approaches will emerge from the better insight into the pathophysiology of NBIA subtypes. For example, our field has recently seen exciting new therapies for related inherited rare disorders such as spinal muscular atrophy and Huntington's disease using antisense oligonucleotides (135, 136). Of these, work in SMA patients has highlighted the role of ASOs in restoring protein expression by modulating splicing (135, 137). This may be helpful in disorders where the majority of genetic defects are splice site mutations. For the moment, however, this does not apply in most NBIA cases, although splice site mutations have been described in individual cases. In HD, ASOs act by reducing the toxic gain of function caused by the autosomal dominant mutation. Again, while this approach may not be feasible for the autosomal recessively inherited NBIA disorders it may be an option for autosomal dominant NBIA subtypes (e.g., neuroferritinopathy) where a healthy allele of the specific gene is present. Currently, a feasible approach for genetic therapy in NBIA disorders seems to be AAV-assisted gene supplementation.

Until the new mechanistic treatments are available for patients, iron removal remains a sensible option. As summarized above, iron chelators have shown the ability to reduce brain iron accumulation and slow disease progression in certain subsets of NBIA patients (i.e., atypical PKAN patients). The induction of TfR1 palmitoylation is another potential uniform therapeutic strategy that, if beneficial, may be helpful in many or some of the NBIA disorders.

Given the neurodegenerative nature of the disorders, it will be pivotal to recruit patients early in the disease course before neuronal damage has led to irreversible functional deficits. With the improvement of diagnostic possibilities for detection of gene mutations, more patients can be successfully diagnosed early but also presymptomatic mutation carriers can be correctly identified. Thus, screening of people at risk (e.g., relatives of an NBIA patient) may open a therapeutic window for preventive interventions. New trial designs which are suitable for small cohorts (of rare disorders) may also help to gain the most information from the available data (138).

## Author Contributions

VI wrote the first draft of the manuscript. All authors contributed to manuscript revision, read, and approved the submitted version.

## Conflict of Interest

TK served as coordinating investigator of the FORT trial; received research funding from Retrophin, Inc.; served as coordinating investigator of the deferiprone in PKAN randomized and extension trial; received research funding from ApoPharma Inc.; received support from the European Commission 7th Framework Programme (FP7/2007-2013, HEALTHF2-2011; Grant Agreement No. 277984, TIRCON) and from the European Reference Network for Rare Neurological Diseases (ERN-RND), co-funded by the European Commission (ERN-RND: 3HP 767231); provided consulting services to CoA Therapeutics, Comet Therapeutics, and Retrophin, Inc.; received travel support from ApoPharma Inc. SS was supported by the LMU Excellence Initiative, Braun Stiftung, Ara Parseghian Medical Research Foundation, and Stiftung Verum. The remaining authors declare that the research was conducted in the absence of any commercial or financial relationships that could be construed as a potential conflict of interest.
